# Lycopene inhibits endothelial‐to‐mesenchymal transition of choroidal vascular endothelial cells in laser‐induced mouse choroidal neovascularization

**DOI:** 10.1111/jcmm.17730

**Published:** 2023-04-17

**Authors:** Lele Li, Xin Cao, Lili Huang, Xiaobo Huang, Jiayi Gu, Xiaoli Yu, Yan Zhu, Yue Zhou, Yu Song, Manhui Zhu

**Affiliations:** ^1^ Department of Ophthalmology Affiliated Hospital 2 of Nantong University Nantong 226001 China; ^2^ Department of Ophthalmology Lixiang Eye Hospital of Soochow University Suzhou 215001 China

**Keywords:** choroidal neovascularization, choroidal vascular endothelial cells, endothelial to mesenchymal transition, lycopene

## Abstract

Choroidal neovascularization (CNV), is a major cause of irreversible blindness among the elderly population in developed countries, which is resulted from subretinal fibrosis without effective therapeutic strategies. Endothelial‐to‐mesenchymal transition (EndMT) of choroidal vascular endothelial cells (CVECs) contributes to subretinal fibrosis. Lycopene (LYC), a non‐pro‐vitamin A carotenoid, plays an anti‐fibrotic role. Herein, we explored the effect and mechanism of LYC on the EndMT of CVECs during CNV. Firstly, LYC inhibited EndMT in hypoxic human choroidal endothelial cells (HCVECs). Meanwhile, LYC inhibited proliferation, androgen receptor (AR) expression and nuclear localization in hypoxic HCVECs. Then LYC‐inhibited AR promotes the activation of microphthalmia‐associated transcription factor (MITF) in hypoxic HCVECs. In addition, LYC down‐regulated AR and induced MITF up‐regulated pigment epithelium‐derived factor (PEDF) transcription and expression in hypoxic HCVECs. Moreover, LYC‐induced PEDF bound to laminin receptor (LR), inhibiting EndMT of hypoxic HCVECs via down‐regulating protein kinase B (AKT)/β‐catenin pathway. In vivo, LYC alleviated mouse laser‐induced subretinal fibrosis secondary to CNV via up‐regulating PEDF without any ocular or systemic toxicity. These results indicate that LYC inhibits EndMT of CVECs via modulating AR/MITF/PEDF/LR/AKT/β‐catenin pathway, showing LYC is a promising therapeutic agent for CNV.

## 1 INTRODUCTION

Choroidal neovascularization (CNV) is a type of fibrovascular proliferation that occurs beneath the central retina (macula). This condition can cause significant vision loss and blindness on a global scale. CNV can be categorized into two types based on the tissues it affects, CNV type I where the tissue penetrates Bruch's membrane, and in CNV type II, the tissue penetrates the monocellular layer of the retinal pigment epithelium (RPE). Both types can ultimately lead to the development of subretinal fibrosis, resulting in permanent damage to photoreceptors and vision loss. The most common cause of CNV in older adults is neovascular age‐related macular degeneration (nAMD). The use of anti‐vascular endothelial growth factor (VEGF) therapy has become a standard practice for stabilizing or improving visual function. Despite frequent intravitreal injections of these agents, many patients experience poor visual outcomes due to subretinal fibrosis.^1^, ^2^ The phenomenon indicates besides VEGF, other factors also participate in the subretinal fibrosis process during CNV.

Endothelial to mesenchymal transition (EndMT) of choroidal vascular endothelial cells (CVECs) contributes to subretinal fibrosis in CNV.^3^ Anti‐fibrotic pigment epithelium‐derived factor (PEDF), also named Serpin family F member 1 (SERPINF1), is transcriptionally promoted by microphthalmia‐associated transcription factor (MITF).^4^ PEDF treatment inhibits the phosphorylation of VEGF downstream signalling partner protein kinase B (AKT) in primate retinal endothelial cells.^5^ Phosphorylation of β‐catenin by AKT increases its transcriptional activity.^6^ Additionally, AKT‐mediated β‐catenin signalling promotes EndMT of human microvascular endothelial cells (HMECs).^7^

Lycopene (LYC), a non‐pro‐vitamin A carotenoid, is found at high concentrations in red fruits and vegetables such as tomatoes and red grapefruit. LYC decreases stellate cell activation to improve hepatic fibrosis in the mouse nonalcoholic steatohepatitis (NASH) model.^8^ Additionally, LYC has been applied for the treatment of oral submucous fibrosis.^9^ However, the role and mechanism of LYC on subretinal fibrosis in CNV are unknown.

Herein, we investigated the effect of LYC on hypoxia‐challenged CVECs in vitro and subretinal fibrosis in a laser‐induced mouse CNV model in vivo. Moreover, we explored the signal pathway involved in the role of LYC. Our results will open a door to a potential therapeutic strategy for CNV.

## 2 MATERIALS AND METHODS

### 2.1 Cell culture and treatments

Human CVECs (HCVECs; CP‐H092 cells from Procell Life Science & Technology Co. Ltd., China) were cultured in DMEM supplemented with 10% foetal bovine serum [FBS; 26,140, Thermo Fisher Scientific (TFS), USA] and 1% penicillin–streptomycin (15,140,122, TFS). HCVECs were grown at either normoxia (20% O_2_, 5% CO_2_ and 75% N_2_ for 48 h; normal) or hypoxia (1% O_2_, 5% CO_2_ and 94% N_2_ for 48 h) in a humidified incubation chamber at 37°C. LYC (SMB00706, Sigma Aldrich, USA) dissolved in 0.1% DMSO (D8418, Sigma Aldrich) at a concentration of 5, 10, 20 or 40 μM of the culture medium of HCVECs was added to HCVECs for 48 h. Testosterone (TES; AR agonist; T1500, Sigma Aldrich), ML329 (MITF inhibitor; S0145, Selleck, USA), SC79 (AKT activator; 305,834–79‐1, MedChemExpress, USA) or SKL2001 (β‐catenin agonist; HY‐101085, MedChemExpress) dissolved in 0.1% DMSO was used at a concentration of 1 μM, 2 μM, 10 μM or 60 μM of the culture medium of HCVECs for 48 h, respectively. Human PEDF recombinant protein (1177‐SF, R&D Systems, USA) was added to HCVECs at the concentration of 2 μg/mL for 48 h.

### 2.2 Western blot (WB)

HCVECs were lysed in ice‐cold RIPA buffer (R0278, Sigma Aldrich) containing protease and phosphatase inhibitor cocktail (PPC1010, Sigma Aldrich). The protein concentrations were quantified utilizing the BCA protein assay kit (71285‐M, Millipore, USA). The sample loading was carried out on SDS‐PAGE gel, and the samples were separated via electrophoresis and then moved to PVDF membranes (IPVH00010, Millipore). The membranes were blocked in TBST containing 5% (w/v) dry skim milk and incubated separately with primary antibodies, including rabbit anti‐cluster of differentiation 31 (CD31) (1:2000, 11265‐1‐AP), mouse anti‐vascular endothelial cadherin (VE‐cadherin) (1:1000, 66804‐1‐Ig), rabbit anti‐mesenchymal cell markers alpha‐smooth muscle actin (α‐SMA) (1:3000; 14395‐1‐AP), mouse anti‐vimentin (1:20,000; 60330‐1‐Ig), rabbit anti‐phosphorylated androgen receptor (p‐AR; S213; 1:500; PA5‐37478, Thermo Fisher Scientific), rabbit anti‐AR (1:500; PA1‐110, Thermo Fisher Scientific), rabbit anti‐phosphorylated microphthalmia‐associated transcription factor (p‐MITF; S180/73; 1:500; SAB4503940, Sigma Aldrich), rabbit anti‐MITF (1:500; HPA003259, Sigma Aldrich), rabbit anti‐pigment epithelium‐derived factor (PEDF; 1:500; 26045‐1‐AP), rabbit anti‐PEDF receptor (PEDF‐R; 1:500; 55190‐1‐AP), mouse anti‐laminin receptor (LR; 1:5000; 67,324‐1‐Ig), mouse phosphorylated‐protein kinase B (p‐AKT; S473; 1:2000; 66444‐1‐Ig), rabbit anti‐AKT (1:2000; 10176‐2‐AP), rabbit anti‐phosphorylated‐β‐catenin (p‐β‐catenin; S552; 1:1000; 9566, Cell Signaling Technology, USA), mouse anti‐β‐catenin (1:5000; 66379‐1‐Ig), rabbit anti‐Snail (1:500; 13099‐1‐AP), rabbit anti‐histone 3 (H3; 1:4000; 17168‐1‐AP), and rabbit anti‐glyceraldehyde‐3‐phosphate dehydrogenase (GAPDH; 1:20,000, 10494‐1‐AP). The signals were detected with secondary antibodies including HRP‐conjugated goat‐anti‐rabbit IgG (1:4000; SA00001‐2) and HRP‐conjugated goat‐anti‐mouse IgG (1:4000; SA00001‐1). Antibodies without any specified suppliers were obtained from Proteintech, USA.

### 2.3 RNA isolation, reverse transcription and quantitative reverse transcription polymerase chain reaction (qRT‐PCR)

Total RNA from HCVECs or mouse retina‐RPE‐choroid complex was isolated using QIAwave RNA mini kit (74536; Qiagen, USA) according to the manufacturers' instructions. Reverse transcription was carried out utilizing the PrimeScript cDNA synthesis kit (6110A; Takara, Japan) after RNA quantitation and DNase I treatment. The qRT‐PCR was carried out utilizing SYBR green supermix (1725272; Bio‐Rad, USA) with primers listed in Table 1.

**TABLE 1:** The information of PCR primers used in the study was shown

Name	Reference sequence	Primer sequence (forward)	Primer sequence (reverse)	PCR Product length (bp)
Human CD31	NM_000442.5	AACGGAAGGCTCCCTTGATG	TAAGAACCGGCAGCTTAGCC	180
Human α‐SMA	NM_001141945.3	AGTTCCGCTCCTCTCTCCAA	ACGCTGGAGGACTTGCTTTT	199
Human PEDF	NM_001329903.2	ATGAACCCTTTCTGCTGCCT	CACCCGGTACAGGTCATAGC	250
Mouse Snail	NM_011427.3	ATGGAGTGCCTTTGTACCCG	CAGTAACCACCCTGCTGAGG	261
Mouse α‐SMA	NM_007392.3	GTACCCAGGCATTGCTGACA	GCTGGAAGGTAGACAGCGAA	146
Human GAPDH	NM_001256799.3	AATGGGCAGCCGTTAGGAAA	GCGCCCAATACGACCAAATC	168
Mouse GAPDH	NM_001289726.2	GCCTCCTCCAATTCAACCCT	TACGGCCAAATCCGTTCACA	140

### 2.4 Detection of cell viability by cell counting kit‐8 (CCK‐8)

The HCVECs from various treatment groups were counted, and their concentration was adjusted to 1 × 10^5^/mL. Subsequently, they were seeded into a 96‐well plate with 100 μL of media in each well. The plates were kept in an incubator for cell culturing at 37°C, 5% CO_2_, for 48 h. Subsequently, CCK‐8 solution (10 μL) has been added to the cells, and the plate was incubated again for 1–4 h. Afterwards, the absorbance (λ 450 nm) was recorded utilizing a plate reader (Multiskan FC, TFS).

### 2.5 Cell transfection

HCVECs reached more than 80% confluence have been utilized for transfection. Lipofectamine RNAiMax transfection agent (6 μL; 13778075, TFS) was mingled with Opti‐MEM (250 μL; 11058021, TFS) and then control siRNA (sc‐37007, Santa Cruz Biotechnology, USA; 1 μM), or human PEDF siRNA (sc‐40947, Santa Cruz Biotechnology; 1 μM) subjected to dilution utilizing Opti‐MEM (250 μL), mixed gently and incubated at room temperature for 20 min. Then, the mixture at the volume of 0.5 mL was introduced into HCVECs in 1.5 mL Opti‐MEM and incubated for 4 h. Afterward, the media was replenished utilizing complete DMEM, followed by subjecting HCVECs to treatments at varied conditions for 48 h.

### 2.6 Immunofluorescence (IF)

HCVECs or mouse choroidal flats plated on gelatin‐coated glass slides were fixed with 4% paraformaldehyde (PFA), permeabilized with 2% bovine serum albumin (BSA; A7030, Sigma Aldrich) and 0.5% Triton X‐100 in PBS, blocked with background buster (NB306, Innovex Biosciences, USA) and incubated overnight at 4°C with primary antibodies anti‐AR, anti‐PEDF, anti‐LR and anti‐α‐SMA, followed by secondary antibodies Alexa Fluor 594 goat‐anti‐rabbit IgG (111‐585‐144, Jackson ImmunoResearch Inc., USA), Alexa Fluor 488 goat‐anti‐mouse IgG (115‐545‐003, Jackson ImmunoResearch Inc.), along with DAPI (C1005, Beyotime, China) staining. Digital images have been acquired utilizing a fluorescence microscope (Leica microsystem, Germany). Co‐localization of AR/DAPI or PEDF/LR was analysed using the included Leica LAS‐AF software.

### 2.7 Sub‐cellular fractionation

HCVECs were fractionated using a cell fractionation kit (ab109719, Abcam, USA) following the manufacturers' protocol. The nuclear and cytoplasmic cell proteins were applied for Western blot to detect the subcellular localization of MITF.

### 2.8 Luciferase reporter assay (LRA)

A MITF binding motif in PEDF's promoter region has been identified utilizing JASPAR (http://jaspar.genereg.net/). Synthesis of wild‐type (WT), as well as mutant (MUT) full‐length human PEDF promoter sequences, was carried out and they were cloned utilizing a pGL3‐basic vector (E1751, Promega, USA). HCVECs were seeded into 24‐well plates and then transfected with 100 ng of pGL3‐PEDF‐WT or pGL3‐PEDF‐MUT via Lipofectamine 3000 (L3000008, TFS). LRA was performed 48 h after transfection and luciferase activities were assessed using the dual‐luciferase reporter assay system (N1610, Promega), according to the manufacturers' instructions.

### 2.9 Chromatin immunoprecipitation (CHIP)‐PCR

The CHIP‐PCR was performed as previous description^10^ using rabbit anti‐PEDF (PA5‐112128, Thermo Fisher Scientific) or rabbit IgG (02–6102, Thermo Fisher Scientific) as the negative control.

### 2.10 Animals and construction of mouse subretinal fibrosis secondary to CNV model

C57BL/6J male mice (purchased from the Laboratory Animal Center of Nantong University) aged between 2 and 3 months were used in the study. All the experimentation proceeded following the ARVO Statement for the Use of Animals in Ophthalmic and Vision Research and was approved by Nantong University Animal Ethics Committee. To induce subretinal fibrosis, on day 1 (the first day), laser‐induced CNV was conducted. Then on day 7, a second laser was applied to each CNV to induce haemorrhage or leakage. Mice were sacrificed 7 days (on day 14) after the second laser treatment.^11^

### 2.11 Animal treatment

The mice were administrated by 0.1% DMSO or LYC via intraperitoneal injection at the dose of 2 mg/kg/day for consecutive 14 days, respectively. Control siRNA (1 μM), PEDF siRNA (sc‐40948, Santa Cruz Biotechnology; 1 μM) or conbercept (CON; KH902; Chengdu Kanghong Biotech Co., China; 1 μL at the concentration of 1 mg/mL) was given to mice via intravitreal injection on day 1 and day 7.

### 2.12 Histological analysis

To investigate the histologic sections of the posterior region of individual mouse eyes, the mice were euthanized and their eyes were removed and fixed overnight in 4% PFA. The eyes were then embedded in paraffin and sliced into sections with a thickness of 5 μm. To analyse the eye sections, haematoxylin and eosin (HE) staining was conducted. The thickness of the retina was determined by measuring the ratio of A (the distance between the ganglion cell layer and the outer edge of the inner nuclear layer) to B (the distance between the ganglion cell layer and the outer edge of the outer nuclear layer).^12^

Mouse major organs including heart, lung, liver, kidney, brain and stomach were fixed in 10% neutral buffered formalin and subjected to further processing procedures of dehydration using graded alcohol and xylene. The histopathological examination was carried out following embedding in paraffin wax. Thereafter, sections (5 μm) have been cut, shifted on glass slides and stained with HE.

### 2.13 Terminal deoxynucleotidyl transferase dUTP nick end labelling (TUNEL) assay

The TUNEL assay was conducted on mouse eye sections embedded in paraffin with the aid of the in situ cell death detection kit and tetramethylrhodamine (TMR) red (12156792910, Roche, Germany), following the manufacturers' guidelines. For total nuclei counterstaining, DAPI was used. The fluorescence microscope was utilized to capture images.

### 2.14 Statistical analysis

Statistical analyses were conducted using GraphPad Prism V5.0 (GraphPad Software, USA). The data were presented as the mean values and the standard error of the mean (SEM). Data analysis was carried out using either one‐ or two‐way anova, followed by Tukey's multiple comparisons test or Student's *t*‐test subsequently. *P* < 0.05 indicated statistical significance.

## 3 RESULTS

### 3.1 LYC inhibits EndMT in hypoxia‐exposed HCVECs

**FIGURE 1:**
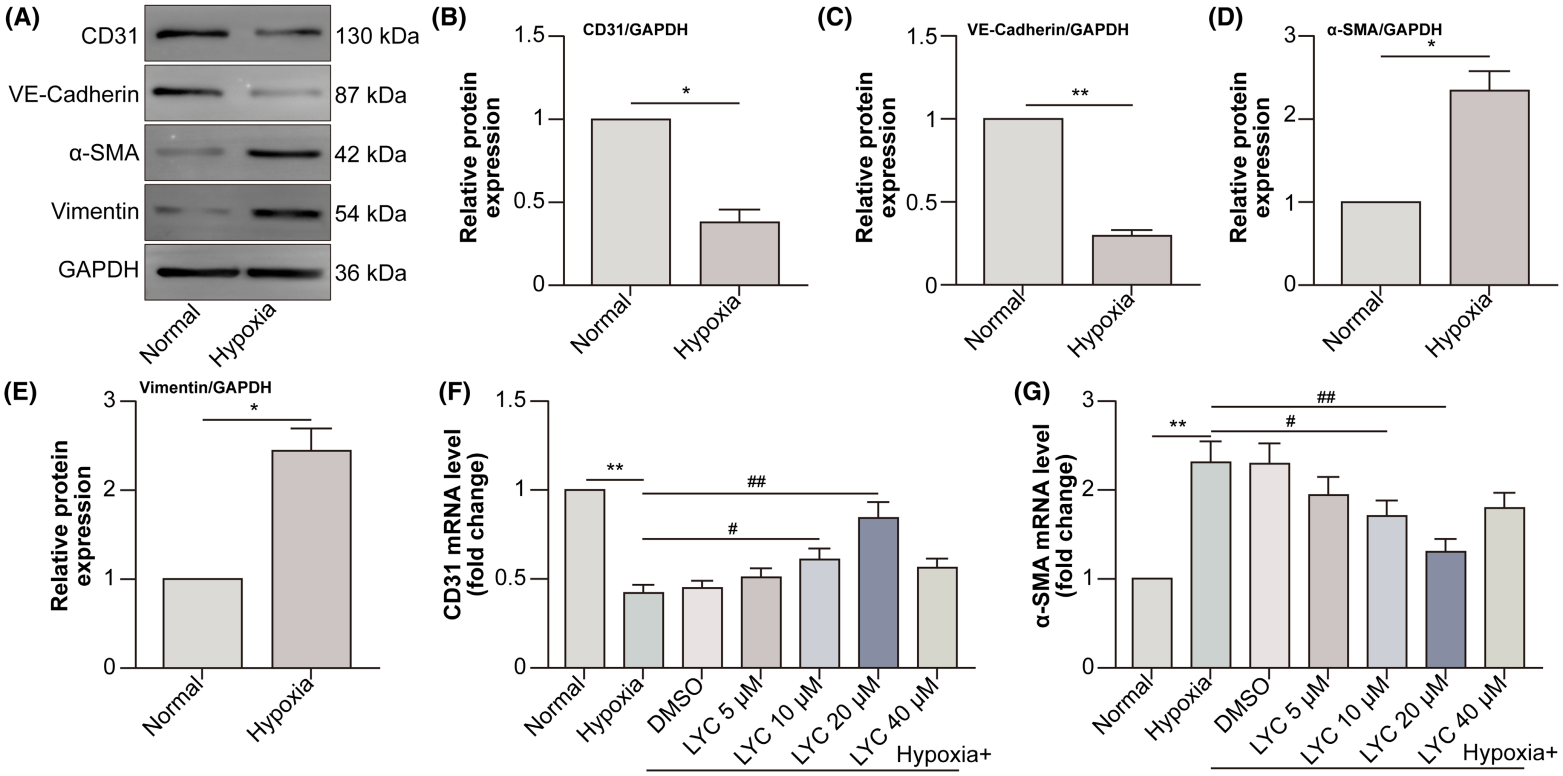
LYC inhibits EndMT in hypoxia‐exposed HCVECs. HCVECs were divided into normal and hypoxia groups. (A) Western blot (WB) is conducted to detect the protein levels of endothelial cell markers CD31 and VE‐cadherin, as well as mesenchymal cell markers α‐SMA and vimentin. (B–E) The relative expression of each molecule i analysed. HCVECs were divided into normal, hypoxia (48 h), hypoxia +0.1% DMSO, hypoxia +5 μM LYC, hypoxia +10 μM LYC, hypoxia +20 μM LYC and hypoxia +40 μM LYC groups. CD31 (F) and α‐SMA (G) mRNA levels were detected by qRT‐PCR. *p < 0.05, **p < 0.01, compared to normal group. ^#^ *p* < 0.05, ^##^ *p* < 0.01, compared to hypoxia group. *n* = 6 in each group.

LYC inhibits epithelial‐mesenchymal transition (EMT) of oral cancer (OC) cells.^13^ However, the effect of LYC on the EndMT of HCVECs is vague. Initially, the expression of endothelial cell markers CD31 and VE‐cadherin with mesenchymal cell markers α‐SMA and vimentin in HCVECs has been identified. The findings revealed that under hypoxic conditions, there was a reduction in the protein levels for endothelial cell markers CD31 and VE‐cadherin, as well as an increase for mesenchymal cell markers α‐SMA and vimentin, in comparison to those in the normal group (Figure 1A–E). Additionally, CD31 mRNA level decreased by hypoxic conditions, while LYC at the dose of 10 μM or 20 μM increased hypoxia‐down‐regulated CD31 mRNA level (Figure 1F). Meanwhile, α‐SMA mRNA level displayed the opposite tendency to CD31 mRNA level (Figure 1G). These data suggested that LYC inhibited EndMT in hypoxic HCVECs.

### 3.2 LYC inhibits proliferation, AR expression and nuclear localization in hypoxia‐exposed HCVECs

**FIGURE 2:**
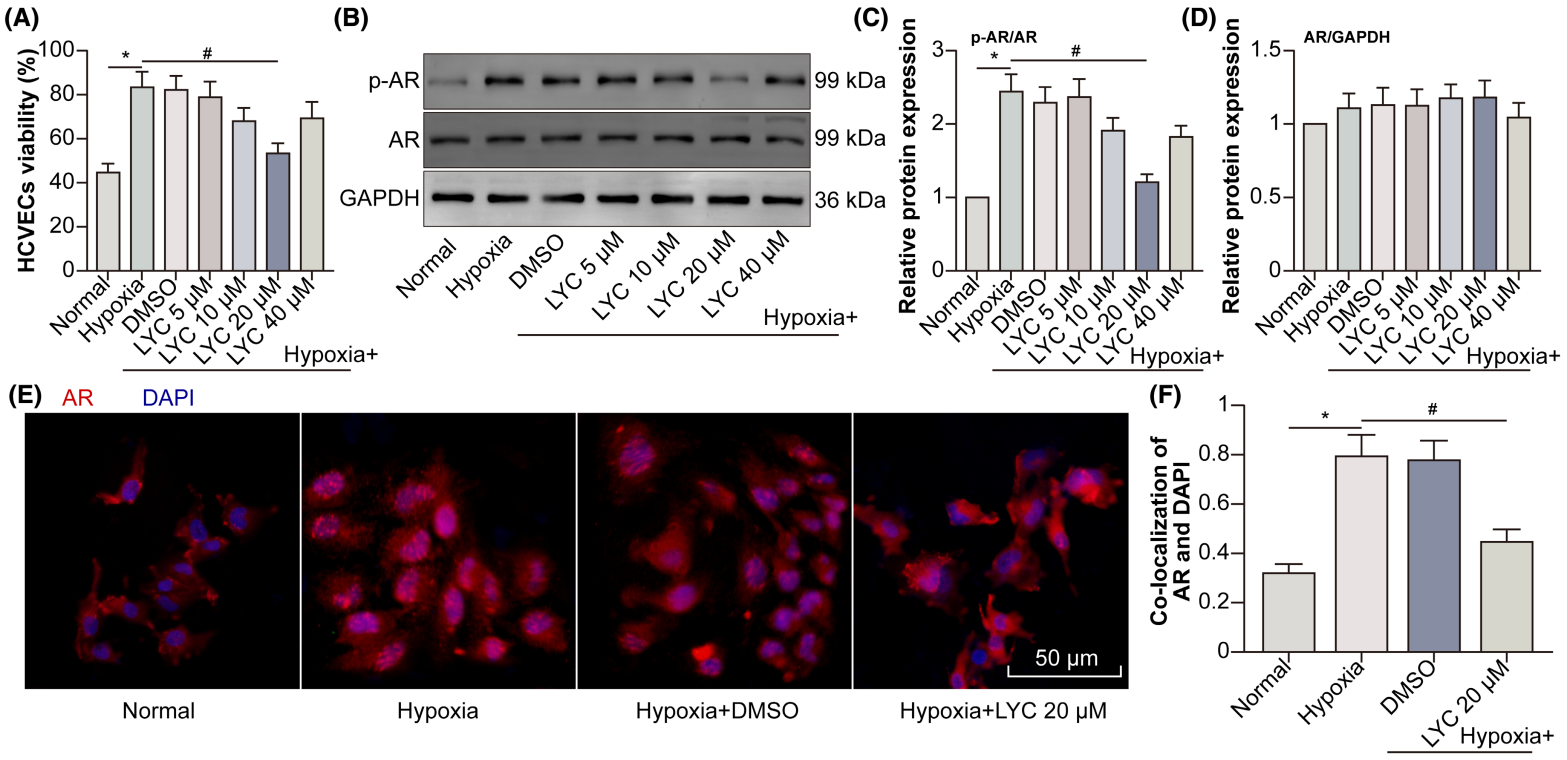
LYC inhibits proliferation, AR expression and nuclear localization in hypoxia‐exposed HCVECs. HCVECs were divided into normal, hypoxia, hypoxia +0.1% DMSO, hypoxia +5 μM LYC, hypoxia +10 μM LYC, hypoxia +20 μM LYC, and hypoxia +40 μM LYC groups. (A) The viability of HCVECs was evaluated by means of CCK‐8 assay. (B) P‐AR and AR protein levels were detected by WB. (C, D) P‐AR and AR protein levels were analysed. (E) AR was detected in normal, hypoxia, hypoxia +0.1% DMSO and hypoxia +20 μM LYC groups by IF. DAPI (blue) was used for labelling the nucleus. (F) The co‐localization of AR as well as DAPI was analysed utilizing the Pearson coefficient (PCC). *p < 0.05, compared to the normal group. ^#^ *p* < 0.05, compared to the hypoxia group. *n* = 5 in each group.

Androgen receptor (AR) had initial localization in the cytoplasm without binding to its ligand. Binding to dihydrotestosterone (DHT), the most active physiological agonist of AR, results in the homodimerization of AR, followed by nuclear translocation and recruitment to the androgen response elements (AREs), triggers transcription and boosts cell proliferation.^14^ LYC inhibits the proliferation and AR expression of LnCaP cells (androgen‐sensitive human prostate adenocarcinoma cells).^15^ Thus, we investigated the effect of LYC on proliferation and AR expression in hypoxic HCVECs. Hypoxia promoted the proliferation of HCVECs when LYC at the dose of 20 μM inhibited the hypoxia‐induced proliferation of HCVECs (Figure 2A). Interestingly, LYC at the dose of 20 μM also down‐regulated hypoxia‐induced p‐AR protein levels with the unchanged tendency of AR protein levels (Figure 2B–D). In addition, LYC at the dose of 20 μM decreased the nuclear localization of AR in HCVECs following hypoxia (Figure 2E,F). The results indicated that LYC inhibited proliferation, AR expression and nuclear localization in hypoxic HCVECs.

### 3.3 LYC‐inhibited AR promotes the activation of MITF in HCVECs under hypoxic conditions

**FIGURE 3:**
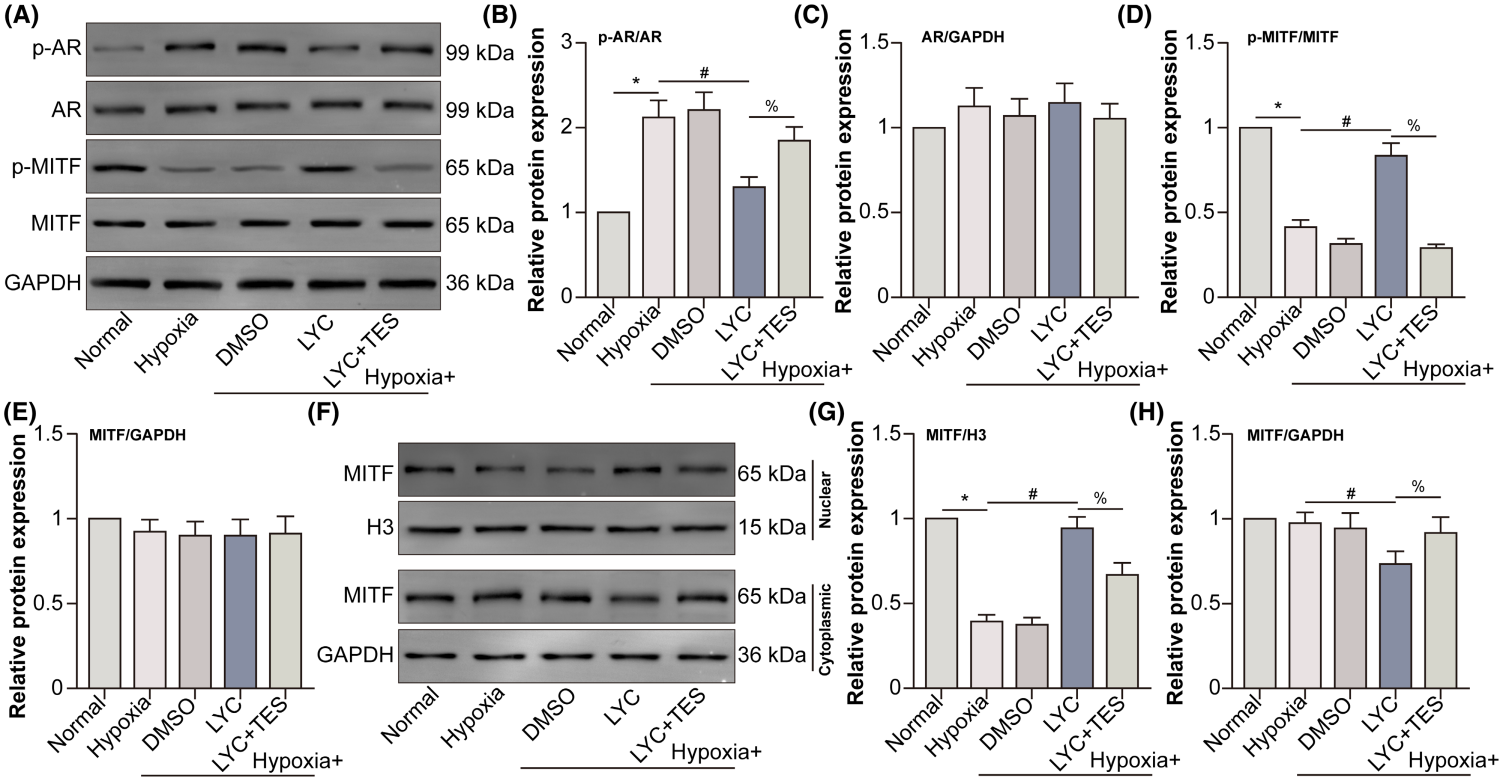
LYC‐inhibited AR promotes the activation of MITF in HCVECs under hypoxic conditions. HCVECs were divided into normal, hypoxia, hypoxia +0.1% DMSO, hypoxia + LYC (20 μM), and hypoxia + LYC + TES groups. (A) P‐AR, AR, p‐MITF and MITF protein levels were detected utilizing WB. (B–E) The relative expression of each molecule was analysed. (F) Nuclear and cytoplasmic separation and WB were done to detect MITF translocation. (G, H) MITF relative expression in the nucleus and cytoplasm was analysed. *p < 0.05, compared to the normal group. ^#^ *p* < 0.05, compared to the hypoxia group. ^%^ *p* < 0.05, compared to the hypoxia + LYC group. *n* = 5 in each group.

Overexpressing AR in melanoma A375 and WM115 cells significantly decreases MITF protein levels.^16^ LYC down‐regulated hypoxia‐induced protein levels of p‐AR in HCVECs, while AR agonist TES partly reversed p‐AR expression. The protein levels of p‐MITF showed the inverse tendency to p‐AR expression, while AR and MITF protein levels had insignificant changes among different groups (Figure 3A–E). Moreover, nuclear translocation of MITF in the hypoxia group decreased compared to that in the normal group. Under hypoxic conditions, LYC increased MITF nuclear translocation, which was alleviated by the combined TES treatment (Figure 3F–H). The results suggested that LYC‐inhibited AR promoted the activation of MITF in hypoxic HCVECs.

### 3.4 LYC down‐regulated AR and induced MITF up‐regulate PEDF transcription and expression in HCVECs under hypoxic conditions

**FIGURE 4:**
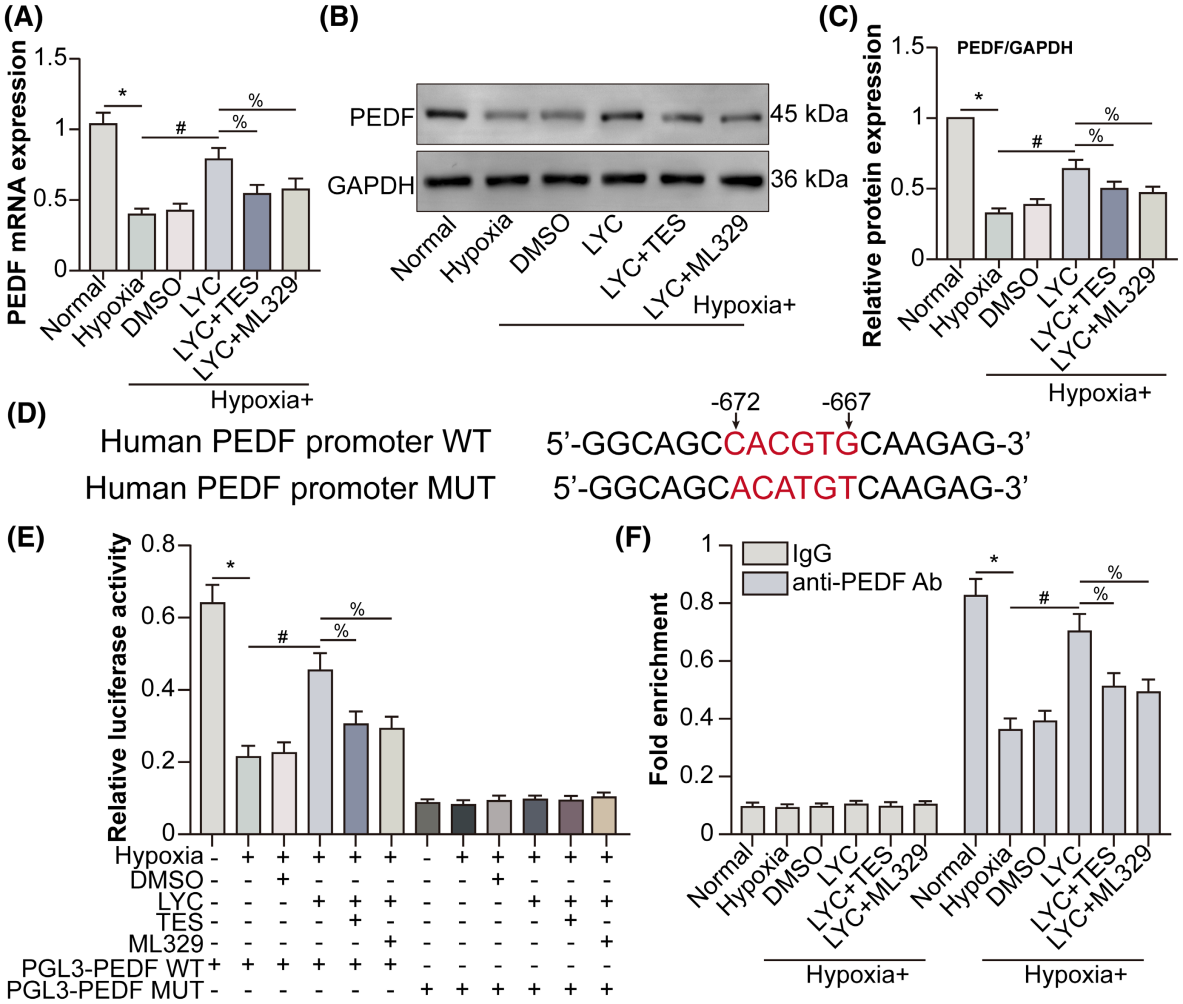
LYC‐down‐regulated AR and ‐induced MITF up‐regulate PEDF transcription and expression in HCVECs under hypoxic conditions. HCVECs have been classified into normal, hypoxia, hypoxia +0.1% DMSO, hypoxia + LYC (20 μM), hypoxia + LYC + TES, and hypoxia + LYC + ML329 groups. (A) PEDF mRNA levels were detected by qRT‐PCR. (B) PEDF protein levels were detected by WB. (C) PEDF protein levels were analysed. (D) The MITF binding site (5′‐CACGTG‐3′, from −672 to −667) in the human PEDF promoter is shown. (E) The activity of the human PEDF promoter was detected by luciferase reporter assay in HCVECs. (F) The direct binding of MITF to the PEDF promoter was detected by CHIP. *p < 0.05, compared to the normal group. ^#^ *p* < 0.05, in comparison to the hypoxia group. ^%^ *p* < 0.05, in comparison to the hypoxia + LYC group. *n* = 5 in each group.

Acting as a transcription factor, MITF up‐regulates PEDF transcription and expression via binding to PEDF RNA in melanoma cells.^17^ The levels of PEDF mRNA as well as protein in HCVECs were found to be decreased by hypoxia. Following hypoxia, LYC promoted PEDF transcription (Figure 4A) and expression (Figure 4B,C), which were partly shifted by TES or MITF inhibitor ML329. Relative luciferase activity assay further notarized the positive regulatory role of LYC on the transcription of PEDF via down‐regulating RA and up‐regulating MITF (Figure 4E). MITF binds to M‐boxes (5′‐TCATGTG‐3′) and symmetrical DNA sequences (E‐boxes) (5′‐CACGTG‐3′) inside the promoters of target genes, such as B‐cell lymphoma 2 (BCL2) and tyrosinase (TYR),^18^ participating in cell differentiation, proliferation and survival.^19^ CHIP assay certified that MITF directly bound to E‐boxes in the promoter of PEDF RNA, whereas LYC enhanced this binding which was down‐regulated by hypoxia via down‐regulating AR and up‐regulating MITF (Figure 4F). The results suggested that LYC down‐regulated AR and induced MITF facilitated PEDF transcription and expression in hypoxic HCVECs.

### 3.5 LYC‐induced PEDF binds to LR on HCVECs under hypoxic conditions

**FIGURE 5:**
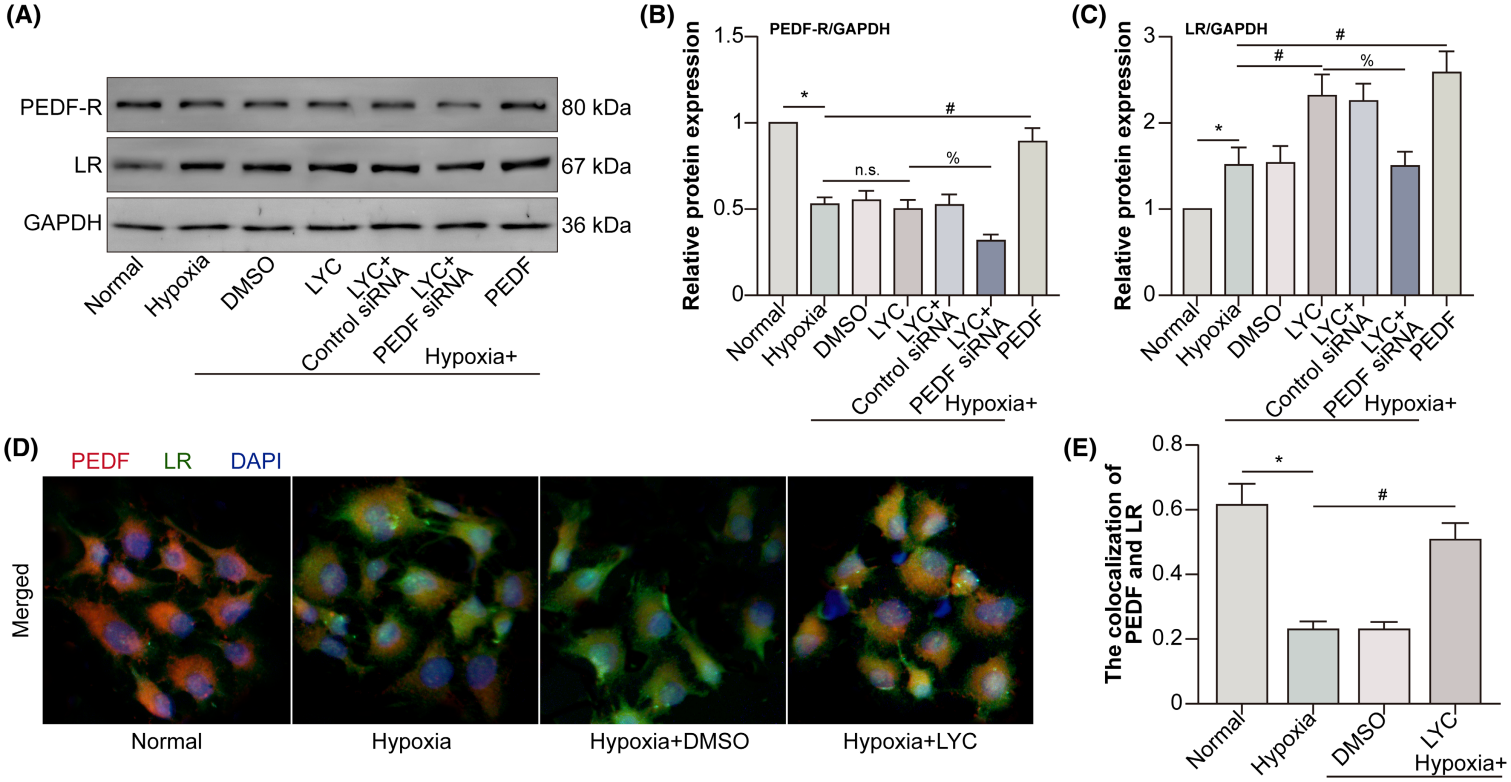
LYC‐induced PEDF binds to LR on HCVECs under hypoxic conditions. HCVECs were divided into normal, hypoxia, hypoxia +0.1% DMSO, hypoxia + LYC (20 μM), hypoxia + LYC + control siRNA, hypoxia + LYC + PEDF siRNA and hypoxia + human PEDF recombinant protein groups. (A) PEDF‐R and LR protein levels were detected by WB. (B‐C) PEDF‐R and LR protein levels were analysed. (D) HCVECs were divided into normal, hypoxia, hypoxia +0.1% DMSO and hypoxia + LYC groups. PEDF and LR were labelled in CECs. (E) The co‐localization of PEDF and LR was analysed. *p < 0.05, in comparison to the normal group. ^#^ *p* < 0.05, in comparison to the hypoxia group. ^%^ *p* < 0.05, in comparison to the hypoxia + LYC group. n.s. denoted no significance. *n* = 5 in each group.

Pigment epithelium‐derived factor receptor (PEDF‐R), also named patatin‐like phospholipase 2 gene product (PNPLA2), expressed on endothelial cells, shows a high affinity for PEDF.^20^ Laminin receptor (LR) is another receptor for PEDF and mediates the angiogenesis inhibitory role of PEDF.^21^ To determine the binding of LYC‐prompted PEDF to which receptor on hypoxic HCVECs, Western blot was performed to measure PEDF‐R and LR expression. Human PEDF recombinant protein was used as the positive control (Figure 5A). Compared with PEDF‐R expression in the normal group, it decreased following hypoxia. LYC did not affect PEDF‐R expression under hypoxic conditions, but PEDF knockdown by PEDF siRNA transfection decreased PEDF‐R expression combined with LYC treatment in the hypoxia group. PEDF overexpression by PEDF recombinant protein promoted PEDF‐R expression following hypoxia (Figure 5B). HCVECs expressed more LR in the hypoxia group than in the normal group. LYC‐elevated hypoxia‐induced LR expression, while simultaneously applying PEDF knockdown partly reduced LR expression. PEDF overexpression also augmented LR expression in hypoxic HCVECs (Figure 5C). Furthermore, fluorescence staining demonstrated that following hypoxia, the co‐localization of PEDF and LR decreased, and LYC increased their co‐localization (Figure 5D,E). The results indicated that LYC induces PEDF to bind to LR on hypoxic HCVECs.

### 3.6 LYC‐induced PEDF inhibits EndMT of HCVECs via down‐regulating AKT/β‐catenin pathway under hypoxic conditions

**FIGURE 6:**
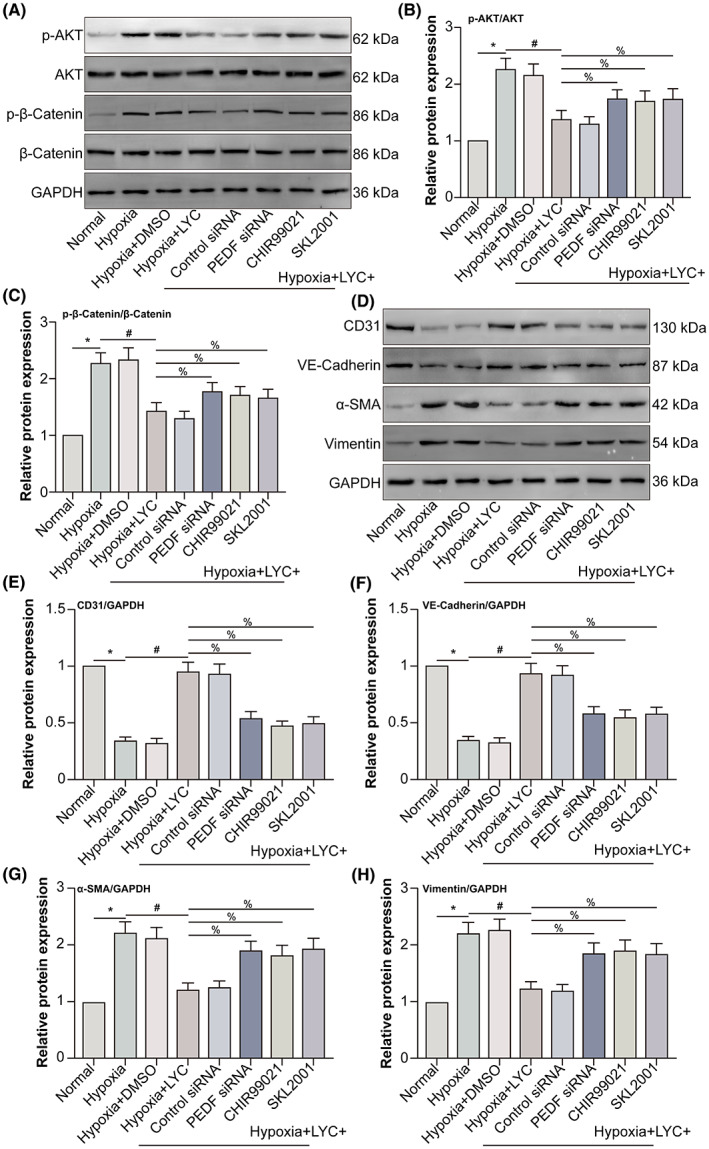
LYC‐induced PEDF inhibits EndMT of HCVECs via down‐regulating AKT/β‐catenin pathway under hypoxic conditions. HCVECs were divided into normal, hypoxia, hypoxia +0.1% DMSO, hypoxia + LYC (20 μM), hypoxia + LYC + control siRNA, hypoxia + LYC + PEDF siRNA, hypoxia + LYC + SC79, and hypoxia + LYC + SKL2001 groups. (A) P‐AKT, AKT, p‐β‐catenin and β‐catenin protein levels were detected by WB. (B, C) The relative expression of p‐AKT and p‐β‐catenin was analysed. (D) CD31, VE‐cadherin, α‐SMA and vimentin protein levels were detected by WB. (E–H) The relative expression of each molecule was analysed. *p < 0.05, in comparison to the normal group. ^#^ *p* < 0.05, in comparison to the hypoxia group. ^%^ *p* < 0.05, in comparison to the hypoxia + LYC group, *n* = 5 in each group.

AKT has been reported to phosphorylate β‐catenin at serine 552 (S552). Phosphorylation at S552 induces β‐catenin accumulation in the nucleus and increases its transcriptional activity.^22^ AKT/β‐catenin pathway increases fibroblast markers α‐SMA as well as collagen I mRNA levels in LX‐2 cells (a human hepatic stellate cell line), indicating that AKT/β‐catenin pathway enhances the activation of LX‐2 cells as well as liver fibrosis.^23^ PEDF overexpression can significantly decrease the phosphorylation of AKT in skeletal myocytes.^24^ To explore the mechanism of PEDF inhibiting EndMT of hypoxic HCVECs, we performed Western blot to measure p‐AKT and p‐β‐catenin expression (Figure 6A). AKT and β‐catenin phosphorylation increased following hypoxia, whereas LYC inhibited hypoxia‐induced AKT and β‐catenin phosphorylation. Additionally, PEDF knockdown via PEDF siRNA transfection or AKT activator SC79 partly reversed the inhibitory role of LYC on hypoxia‐induced AKT phosphorylation, which wasn't affected by β‐catenin agonist SKL2001 (Figure 6B). Unlike AKT phosphorylation, PEDF knockdown, AKT activator SC79 or β‐catenin agonist SKL2001 partly reversed the inhibitory role of LYC on hypoxia‐induced β‐catenin phosphorylation (Figure 6C). Additionally, Western blot was done to detect endothelial and mesenchymal cell markers in HCVECs (Figure 6D). The expression of endothelial cell markers CD31 and VE‐Cadherin decreased, whereas the expression of mesenchymal cell markers α‐SMA, as well as vimentin, increased in the hypoxia group in contrast with the normal group. In hypoxic HCVECs, LYC was found to promote the expression of endothelial cell markers and inhibit the expression of mesenchymal cell markers. PEDF knockdown, AKT activator SC79 or β‐catenin agonist partly shift the roles of LYC on the expression of endothelial and mesenchymal cell markers in hypoxic HCVECs (Figure 6E–H). The results suggested that LYC‐induced PEDF inhibited EndMT of hypoxic HCVECs via down‐regulating AKT/β‐catenin pathway.

### 3.7 LYC alleviates subretinal fibrosis via up‐regulating PEDF in laser‐induced mouse CNV model

**FIGURE 7:**
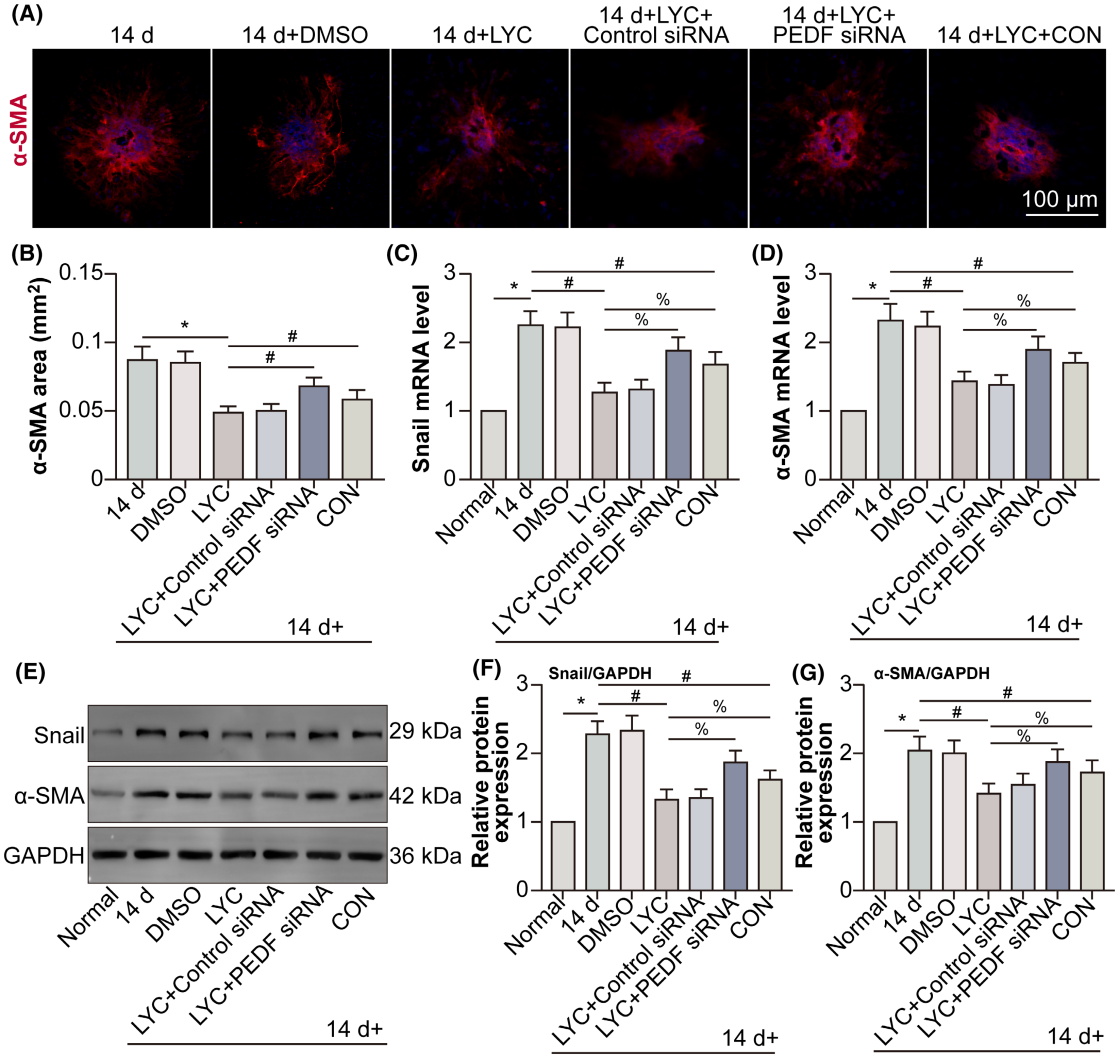
LYC alleviates mouse laser‐induced subretinal fibrosis secondary to CNV via up‐regulating PEDF. The mice were divided into normal, CNV 14 days, CNV 14 days + 0.1% DMSO (oral gavage; 0.8 mg/kg/day for consecutive 14 days), CNV 14 days + LYC (oral gavage; 0.8 mg/kg/day for consecutive 14 days), CNV 14 days + LYC + control siRNA, CNV 14 days + LYC + PEDF siRNA, and CNV 14 days + CON groups. (A) Representative images of subretinal fibrosis lesions by marking α‐SMA in each group (except the normal group) at post‐laser 14 days were shown. Scale bar = 100 μm. (B) Quantitative analyses for the area of α‐SMA were done. Snail (C) and α‐SMA (D) mRNA levels were detected by qRT‐PCR. (E) Snail and α‐SMA protein levels were detected by Western blot. Snail (F) and α‐SMA (G) protein levels were analysed. *p < 0.05, in comparison to the normal group. ^#^ *p* < 0.05, in comparison to the hypoxia group. ^%^ *p* < 0.05, in comparison to the hypoxia + LYC group. *n* = 5 in each group.

To seek the role and mechanism of LYC on subretinal fibrosis during CNV, mouse laser‐induced subretinal fibrosis secondary to the CNV model was constructed and PEDF siRNA was administrated by intravitreal injection to knockdown PEDF. α‐SMA reactivity was decreased by LYC following CNV, and PEDF knockdown partly reversed the negative regulatory role of LYC on α‐SMA reactivity. Conbercept (CON) is a recombinant fusion protein possessing key domains 2, 3 and 4 from VEGF receptors 1 and 2, blocking VEGF signalling. In December 2013, CON has been approved by the China State Food and Drug Administration for the treatment of nAMD.^25^ Therefore, CON was applied as the positive therapeutic control. At the same time, CON intravitreal injection also decreased α‐SMA reactivity following CNV, but the efficiency of LYC was more than that of CON (Figure 7A,B). Additionally, the transcription as well as expression of the key transcription factor, Snail, which is a well‐known inducer of EndMT in hypoxia‐induced human coronary endothelial cells^26^ and α‐SMA were examined by means of qRT‐PCR and Western blot, respectively. The Snail, as well as α‐SMA mRNA levels, were found to be higher in the CNV 14 days group compared to those in the normal group. Following CNV, LYC down‐regulated Snail and α‐SMA mRNA levels, which were partly increased by combined PEDF knockdown. CON decreased Snail and α‐SMA mRNA levels in the CNV group. However, LYC showed a stronger effect than that of CON (Figure 7C,D). Snail and α‐SMA protein levels displayed a similar tendency as their mRNA levels (Figure 7E–G). The results suggested that LYC mitigated subretinal fibrosis via up‐regulating PEDF in mouse CNV.

### 3.8 LYC causes no ocular or systemic toxicity

**FIGURE 8:**
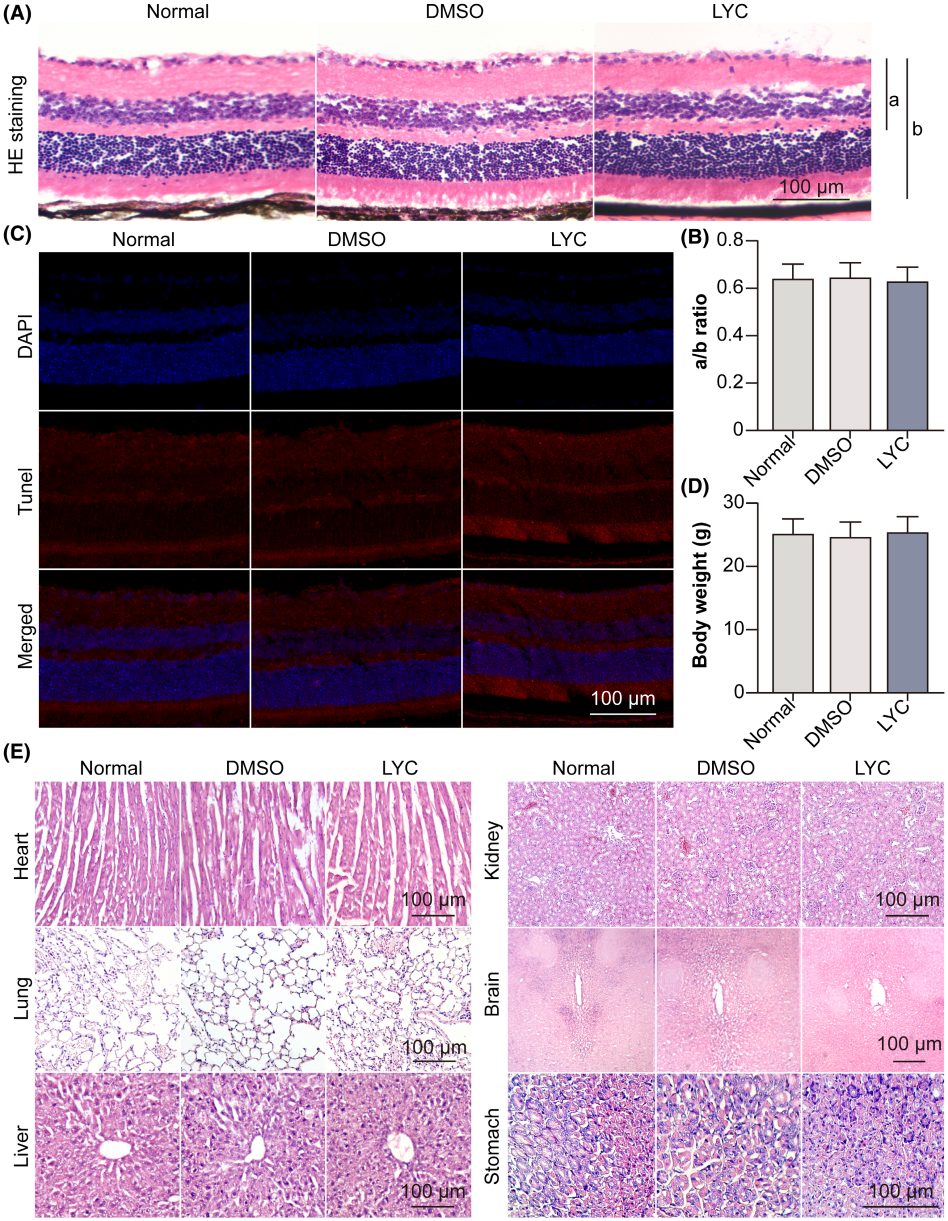
LYC causes no ocular or systemic toxicity. The mice were divided into normal, 0.1% DMSO (oral gavage; 0.8 mg/kg/day for consecutive 14 days), and LYC (oral gavage; 0.8 mg/kg/day for consecutive 14 days) groups. (A) HE staining on retina‐RPE‐choroid cryosection was done. (B) The A/B ratio was analysed. (C) TUNEL staining on retina‐RPE‐choroid cryosection was done. (D) The body weight of each mouse was measured. (E) HE staining on mouse heart, lung, liver, kidney, brain and stomach tissue was done. *n* = 4 in each group.

**FIGURE 9:**
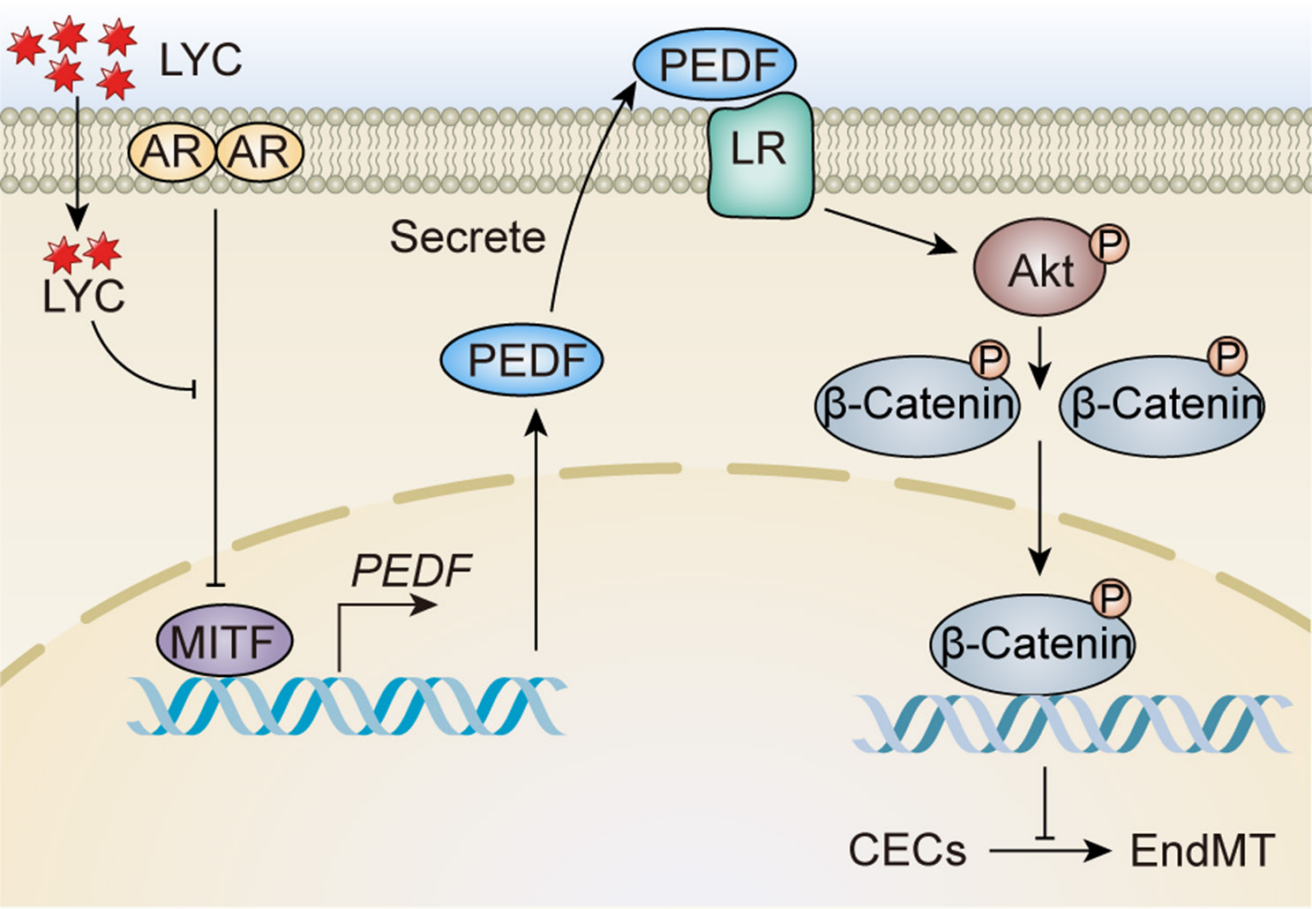
The mechanism diagram of LYC on subretinal fibrosis during CNV is shown.

Finally, the ocular and systemic safety of LYC in normal mice was examined. Histological analysis of HE‐stained tissue sections showed that the retinal tissues in the group that received LYC oral gavage did not exhibit any alterations in histological morphology or retinal thickness when compared to the normal or DMSO oral gavage groups (Figure 8A,B). Moreover, there was no increase in TUNEL‐positive cells in all retinal layers after LYC oral gavage at 14 days compared to that of normal or DMSO oral gavage groups (Figure 8C,D). No degeneration, necrosis or structural change was observed in mouse heart, lung, liver, kidney, brain or stomach tissue section in the LYC oral gavage group compared to that of normal or DMSO oral gavage groups (Figure 8E). The data demonstrated that oral gavage LYC at the dose of 0.8 mg/kg/day for consecutive 14 days was safe to mouse retina and other vital organs. In brief, our study revealed that exogenous LYC up‐regulated MITF via inhibiting AR, hence promoting PEDF secreted from CVECs. The binding between PEDF and LR on CVECs increases, inhibiting the AKT/β‐catenin pathway and EndMT of CVECs (Figure 9).

## 4 DISCUSSION

Subretinal fibrosis during CNV is a prominent characteristic of nAMD, leading to irreversible loss of central vision due to anatomic and functional damage to the retinal pigment epithelium (RPE) as well as photoreceptor cells.^27^ The present study provided crucial information about the significant impact and mechanism of LYC on subretinal fibrosis during CNV. In the study, LYC inhibited hypoxia‐induced EndMT of HCVECs. LYC suppressed hypoxia‐induced proliferation, the expression and nuclear localization of AR in HCVECs, whereas LYC‐inhibited AR promoted the activation of MITF in hypoxic HCVECs. Moreover, LYC‐down‐regulated AR and ‐induced MITF up‐regulated PEDF transcription and expression in hypoxic HCVECs. LYC‐induced PEDF bound to LR on hypoxic HCVECs, inhibiting EndMT of hypoxic HCVECs via down‐regulating AKT/β‐catenin pathway. In vivo, LYC alleviated mouse laser‐induced subretinal fibrosis secondary to CNV via up‐regulating PEDF without ocular or systemic toxicity.

Cobalt chloride (CoCl_2_; a commonly used hypoxia‐mimetic agent; 300 μmol/L for 24 h) increases the expression of mesenchymal markers α‐SMA, vimentin and Snail, as well as decreases expression of endothelial cell markers CD31 and tight junction protein 1 (ZO‐1) in (HUVECs).^28^ Our study revealed that hypoxic conditions (1% O_2_, 5% CO_2_ and 94% N_2_ for 48 h) induced the expression of mesenchymal markers CD31 and VE‐cadherin while inhibiting the expression of endothelial cell markersα‐SMA and vimentin. The data indicated that the EndMT of HCVECs was induced by hypoxia. Both EMT and EndMT are physiological processes that are essential for normal embryogenesis. Under pathological conditions, such processes may be hijacked to promote tissue fibrosis. EMT as well as EndMT are critical for the pathogenesis of subretinal fibrosis in the eye. LYC down‐regulates the expression of EMT markers including Twist family BHLH transcription factor 1 (TWIST) and Snail in ovarian cancer cell‐borne mice compared to those receiving placebo treatment.^29^ Our study showed LYC inhibited EndMT of HCVECs under hypoxic conditions.

Endothelial cell proliferation is positively related to EndMT.^30^ LYC notably inhibits the proliferation of human peripheral blood mononuclear cells^31^ and SKOV3 cells (a human ovarian adenocarcinoma cell line).^32^ Data from the present study show that LYC inhibits the hypoxia‐induced proliferation of HCVECs. AR inhibits EMT of PC‐3 prostate cancer cells.^33^ Furthermore, our study has found that LYC inhibits the expression and nuclear translocation of AR in HCVECs under hypoxic conditions. However, LYC up‐regulates AR mRNA levels in the epididymis of aroclor 1254 (A1254; a commercial mixture of environmental pollutants polychlorinated biphenyls)‐exposed adult rats.^34^ Another study reports that LYC enhances the expression of AR in Leydig cells (testicular cells responsible for the biosynthesis and secretion of androgens).^35^ The differences between the pro‐ and anti‐AR role of LYC might attribute to the instinct cell types and discrepant treatments.

A previous study has demonstrated that AR is important in promoting the proliferation of melanoma by down‐regulating MITF.^36^ MITF is up‐regulated in senescent vascular endothelial cells.^37^ The suppression of miR‐218 alleviates cardiac fibrosis and cardiac function impairment, and stimulates angiogenesis in myocardial infarction (MI) rats through inhibiting MITF,^38^ indicating that MITF promotes cardiac fibrosis. Therein, our study revealed that LYC up‐regulated the phosphorylation of MITF, ultimately inhibiting EndMT of hypoxic HCVECs, demonstrating that MITF might play a role of anti‐fibrosis in CVECs under hypoxic conditions.

PEDF inhibits cardiac fibrosis especially perivascular cardiac fibrosis by regulating EndMT via down‐regulating the transcriptional activation of β‐catenin in an ischemic heart.^39^ Additionally, the immunoreactivity for PEDF is weak in the new vessels where fibrosis was prominent (clinical quiescent choroidal neovascular membranes),^40^ implying the anti‐fibrotic role of PEDF in CNV. In the study, LYC exerted its anti‐fibrotic function via up‐regulating PEDF in HCVECs.

Immunofluorescence demonstrates that human aortic endothelial cells express LR.^41^ PEDF inhibits the expression of fibronectin via LR in MDA‐MB‐231 cells (a human breast cancer cell line),^42^ while fibronectin is one of the extracellular matrix (ECM) components contributing to a dense fibrotic scar of CNV.^43^ The data suggest that PEDF might inhibit subretinal fibrosis via binding to LR, which is consistent with our study. PEDF‐R acts as another receptor for PEDF. As of now, no clear proof exists for the anti‐angiogenic effects of PEDF being mediated by PEDF‐R in endothelial cells. However, it has been demonstrated that PEDF induces the expression of apoptotic Fas ligand (FasL) by regulating nuclear factor kappa B (NF‐κB) in endothelial cells,^44^ and the regulation of NF‐κB by PEDF is PEDF‐R‐dependent.^45^ Hence, it is plausible that PEDF may exert its anti‐angiogenic effects via PEDF‐R in endothelial cells. But the study showed that PEDF did not bind to PEDF‐R on HCVECs under hypoxic conditions.

Akt/β‐catenin axis promotes tubulointerstitial fibrosis via up‐regulating Snail expression.^46^ Nuclear β‐catenin assembles a transcriptionally active complex via recruitment of transcriptional coactivators cyclic AMP response element binding protein (CBP) to stimulate the transcription of target genes. PRI‐724, a selective inhibitor of β‐catenin/CBP displays anti‐fibrotic activity in the hepatitis C virus‐induced mouse liver fibrosis model.^47^ Down‐regulated β‐catenin mediated‐detailed mechanisms caused by LYC on subretinal fibrosis desiderate further investigation.

Until now, several clinical trials have proved that LYC has the potential of preventing or treating multiple diseases. In a randomized controlled clinical trial, it is found that administration of LYC (30 mg 12 h prior to percutaneous coronary intervention and 15 mg prior while 8 h following procedure) considerably lowers the serum creatine kinase‐MB (CK‐MB) levels in 28 patients with MI after undergoing standard treatments with aspirin, statin and β‐blocker.^48^ The findings suggest that LYC has the potential to prevent cardiovascular events following myocardial ischemia–reperfusion (I/R) injury. Furthermore, LYC has been observed to reduce C‐reactive protein (CRP) levels among patients with heart failure as well as healthy individuals.^49^, ^50^ In a human study, 20 metastatic HRPC (hormone‐refractory prostate cancer) patients receiving LYC (10 mg) daily for 3 months have a lower grade of cancer and reduced bone pain.^51^ The anti‐angiogenesis function of LYC in prostate cancer is also proved by a prospective study.^52^ Therefore, LYC might be used for the treatment of CNV.

Certainly, there were several weaknesses in our study. High throughput tests such as mRNA sequencing or transcriptome detection to analyse the overall effects of LYC on hypoxic HCVECs were not carried out. Additionally, the downstream pathway of β‐catenin regulated EndMT of CVECs calls for future exploration. However, our study found the anti‐fibrotic capability of LYC and its mechanisms, expanding the potential of LYC in the treatment of nAMD.

## AUTHOR CONTRIBUTIONS

**Lele Li:** Conceptualization (lead); data curation (lead); resources (equal); writing – original draft (equal); writing – review and editing (equal). **xin Cao:** Data curation (equal); formal analysis (equal); resources (equal). **lili huang:** Data curation (equal); formal analysis (equal); software (equal). **xiaobo huang:** Data curation (equal); formal analysis (equal); supervision (equal). **jiayi gu:** Data curation (equal); formal analysis (equal); validation (equal). **xiaoli yu:** Investigation (equal); methodology (equal); visualization (equal). **yan zhu:** Investigation (equal); methodology (equal); visualization (equal). **yue zhou:** Investigation (equal); methodology (equal); visualization (equal). **Yu Song:** Project administration (lead); writing – original draft (equal). **Manhui Zhu:** Funding acquisition (lead); project administration (lead); writing – original draft (lead); writing – review and editing (lead).

## FUNDING INFORMATION

The study was supported by the National Natural Science Foundation of China (No. 802201219), the Suzhou Science and Technology Bureau (No. SKJYD2021044) and Gusu Health Talent Program Project in Suzhou (No. (2022)192).

## CONFLICT OF INTEREST STATEMENT

All of the authors declare that there is no conflict of interests.

## Data Availability

The data used to support the findings of this study are available from the corresponding author upon reasonable request.
